# Biventricular centrimag support for patients in end-stage biventricular heart failure

**DOI:** 10.1186/1749-8090-8-S1-O146

**Published:** 2013-09-11

**Authors:** V Horvath, P Nemec, J Ondrasek, H Bedanova, J Slavik, P Pokorny, P Pavlik, M Orban

**Affiliations:** 1 International Clinical Research Centre, St. Anne´s University Hospital, Brno, Czech Republic; 2 Centre for Cardiovascular and Transplant Surgery, Brno, Czech Republic

## Background

The CentriMag mechanical support device (Levitronix LLC, Waltham, USA) is intended for short-term paracorporeal circulatory support in patients in refractory cardiogenic shock. The paper presents our experience with this device allowing urgent initiation of biventricular circulatory support.

## Methods

From December 2009 through October 2012, the CentriMag device was implanted in 11 patients (2 women) transferred to our institution with refractory biventricular end-stage heart failure and multisystem organ failure. The mean age of our patients was 46.5 ± 12.0 years (range, 20–64 years). The basic cardiac disease was dilated cardiomyopathy (7 patients), myocarditis (2 patients) and coronary artery disease (2 patients).

## Results

The mean ventricular support time was 26.9 ± 16.5 days (range, 11 –71 days). In six patients (55%), mechanical support was completed with heart transplantation. Recovery of heart function occurred in two (18%) patients. Three (27%) patients died while on the support device. The 30-day and one-year survival rates were 64% (7 patients) and 55% (6 patients), respectively. The mean time from support device implantation to putting the patient on the waiting list was 13.4 ± 20.5 days (range, 0–57 days) depending on recovery of organ (including the CNS) function. The mean waiting time was 14.6 ± 11.3 days (range, 3–35 days). The three-month and one-year survival rates after heart transplantation were 83% and 67% (4 of 6 patients), respectively.

## Conclusion

The installation of the Levitronix CentriMag device in our center resulted in a marked improvement of an otherwise grim prognosis of patients whose anticipated survival rates, unless undergoing urgent mechanical cardiac support implantation would be in the order of hours, or several days at most.

